# Narcissism and Exercise Addiction: The Mediating Roles of Exercise-Related Motives

**DOI:** 10.3390/ijerph18084243

**Published:** 2021-04-16

**Authors:** Virgil Zeigler-Hill, Avi Besser, Maor Gabay, Gracynn Young

**Affiliations:** 1 Department of Psychology, Oakland University, Rochester, MI 48309, USA; gracynnyoung@oakland.edu; 2 Department of Communication Disorders/School for Sciences, Health and Society, Hadassah Academic College, Jerusalem 9101001, Israel; 3 Department of Physical Education and Sports, Kaye Academic College of Education, Be’er Sheva 8414201, Israel; maorgabaygs@gmail.com

**Keywords:** narcissism, exercise, motivation, addiction

## Abstract

The present research examined whether the associations that narcissistic personality features had with exercise addiction were mediated by particular motives for engaging in exercise in a large Israeli community sample (*N* = 2629). The results revealed that each aspect of narcissism was positively associated with exercise addiction. Narcissistic admiration and narcissistic rivalry had similar positive indirect associations with exercise addiction through the interpersonal motive for exercise. However, these aspects of narcissism diverged in their indirect associations with exercise addiction through psychological motives, body-related motives, and fitness motives for exercise such that these indirect associations were *positive* for narcissistic admiration but *negative* for narcissistic rivalry. Narcissistic vulnerability had positive indirect associations with exercise addiction through body-related motives and fitness motives that were similar to those observed for narcissistic admiration. These results suggest that exercise-related motives may play important roles in the associations that narcissistic personality features have with exercise addiction. The discussion will focus on the implications of these results for understanding the complex connections between narcissism and exercise addiction.

## 1. Introduction

Exercise refers to planned, structured, and repetitive physical activities that are intended to improve or maintain some aspect of physical fitness [1,2,3]. Regular physical exercise has been shown to contribute to both physical health (e.g., reduced risk of cardiovascular disease and diabetes) and psychological well-being (e.g., reduced symptoms of depression and anxiety) which has led to the recommendation that nearly everyone should include at least some level of exercise in their lives [4,5,6]. However, some individuals may become so focused on exercise that it can actually have negative consequences for them due to issues such as prioritizing exercise to the extent that other areas of life are neglected [7,8,9,10,11,12]. This has led to concerns that exercise may constitute an addiction for some individuals [13,14,15,16,17]. The purpose of the present research was to examine whether narcissistic personality features were associated with exercise addiction and whether exercise-related motives mediated these associations.

Exercise addiction was initially considered to be relatively harmless given the array of benefits that are associated with exercise [18]. However, the perception of exercise addiction changed as it became clear that exercise addiction was often detrimental to both physical health (due to issues such as overtraining) and psychological functioning (due to issues such as prioritizing exercise over developing and maintaining interpersonal relationships [16]). Exercise addiction differs from simply being highly committed to exercise in various ways including that individuals who are simply *committed* to exercising tend to engage in exercise because they enjoy the benefits of doing so (e.g., they feel better when they are engaging in regular exercise), whereas those who are *addicted* to exercising are more often motivated by a sense of obligation (e.g., they exercise because they anticipate negative consequences if they fail to do so; [9,19]). Furthermore, exercise addiction is characterized by compulsive tendencies and feelings of dependence which are not commonly observed among those who are simply committed to exercise [9]. These differences may explain why those who are addicted to exercise consider exercise to be a central part of their lives and report experiencing powerful feelings of deprivation—which are similar in many respects to withdrawal symptoms—when they are unable to exercise [9]. It has been suggested that exercise addiction may have features that are similar to other addictions such as salience, mood modification, tolerance, and withdrawal symptoms [9].

The cause of exercise addiction remains unclear but various explanations have been offered, including those based on psychophysiological processes such as the production of endorphins [8]. Estimates for the prevalence rate of exercise addiction have varied across studies but it appears to be approximately 3% in the general population [8,11,20,21]. It is important to note that there has been considerable debate about whether exercise addiction should actually be considered as an addiction. For example, exercise addiction is not included as a disorder in the *Diagnostic and Statistical Manual of Mental Disorders* (*DSM-5* [22]). In fact, the only form of addiction included in the *DSM-5* that does not involve the ingestion of a substance is gambling disorder [1]. In addition, there has been an ongoing debate regarding whether exercise addiction should be considered as a standalone disorder or simply as an aspect of eating disorders [23,24,25]. For example, excessive exercise behavior is included as a compensatory behavior for bulimia nervosa in the *DSM-5* [22]. However, we believe that exercise addiction is an important issue that warrants empirical attention, even though it is not currently recognized as a standalone disorder.

### 1.1. Narcissism and Exercise Addiction

The detrimental consequences of exercise addiction suggest that it is important to identify some of its risk factors. One possible risk factor for exercise addiction is narcissism, which refers to a complex and dynamic system of social, cognitive, and affective self-regulatory processes that are characterized by features that include a sense of grandiosity, vanity, self-absorption, feelings of entitlement, a lack of empathy for others, and a willingness to exploit others [26,27,28,29]. Initial support for the connection between narcissism and exercise addiction has been observed [1,30], which has led to speculation that narcissism may be one of the key personality features for predicting exercise addiction [31]. The observed connections between narcissism and exercise addiction are consistent with other research showing narcissism to be associated with a range of addiction-related issues including problematic substance use [32,33,34,35,36] and gambling [32,37,38,39]. In addition, narcissism has been shown to be associated with elevated levels of exercise behaviors [40] and appearance-related concerns [41,42], which are consistent with the possibility that it may serve as a risk factor for exercise addiction.

Narcissism has been found to be associated with exercise addiction but the underlying mechanism that is responsible for this connection remains unclear. One possible mechanism may be particular motives that are related to exercise. This possibility is consistent with previous arguments that motivational factors may mediate the associations that personality traits have with a wide array of attitudes and behaviors [43]. The basic idea is that personality traits may activate motives that are then satisfied by adopting certain attitudes or engaging in particular behaviors. Support for the argument that personality traits are associated with various motives for participating in activities has been observed for a range of behaviors including exercise [44,45,46,47]. Furthermore, exercise-related motives have been found to mediate the associations that basic personality dimensions have with exercise-related behaviors [47]. For example, neuroticism was found to be associated with motives concerning the improvement of appearance and regulation of weight which, in turn, were associated with engagement in exercise. Exercise-related motives have also been shown to be associated with exercise addiction [48,49,50,51] but no studies have examined whether the associations between narcissism and exercise addiction are mediated by exercise-related motives.

### 1.2. Overview and Predictions

The purpose of the present research was to gain a more complete understanding of the associations that narcissistic personality features have with exercise addiction as well as the possibility that exercise-related motives may mediate these associations. Previous studies have examined the connections that narcissism has with exercise-related attitudes and behaviors [1,30] but these studies have often conceptualized narcissism as being unidimensional even though the evidence is abundantly clear that it is actually a multidimensional construct [52,53]. To address this limitation, we adopted the Narcissistic Admiration and Rivalry Concept (NARC [54]) to provide a more nuanced account of the connections that narcissistic personality features have with exercise-related motives and exercise addiction. The NARC model was developed with the intention of clarifying some of the conflicting results that have been previously observed in research concerning narcissism (e.g., narcissistic individuals are considered to be charming by others but they are also perceived as aggressive). The NARC model attempts to resolve some of these issues by distinguishing between *narcissistic admiration* (an agentic aspect of narcissism that is characterized by assertive self-enhancement and self-promotion) and *narcissistic rivalry* (an antagonistic aspect of narcissism that is characterized by self-protection and self-defense). Narcissistic admiration and narcissistic rivalry represent two different strategies for maintaining grandiose self-views.

The NARC model has been extremely useful for clarifying some of the confusion and inconsistencies surrounding the connections that narcissism has with various attitudes and behaviors. For example, narcissistic admiration and narcissistic rivalry have both been shown to be similar in terms of their motivation to attain status but these aspects of narcissism are linked with different strategies for pursuing status [55]. More specifically, narcissistic admiration tends to be characterized by agentic approaches to the pursuit of status that focus on earning prestige, whereas narcissistic rivalry is characterized by the exclusive use of antagonistic strategies for the pursuit of status that involve the use of dominance and intimidation. However, it is important to note that the NARC model is not the only model that is used to distinguish between different aspects of narcissism. In fact, there is an emerging consensus regarding the existence of three distinct aspects of narcissism [27,53,56]. The first aspect is *assertive/extraverted* narcissism which is a purely grandiose form of narcissism that is largely consistent with narcissistic admiration from the NARC model. The second aspect is *antagonistic/disagreeable* narcissism which is a blend of the grandiose and vulnerable forms of narcissism that is largely consistent with narcissistic rivalry from the NARC model. The third aspect is *vulnerable/neurotic* narcissism which is a purely vulnerable form of narcissism that is characterized by negative affectivity and psychological distress. This aspect of narcissism is characterized by a heightened sensitivity to potentially threatening events (e.g., comments from others that may be interpreted as criticisms or insults) which has led to vulnerable/neurotic narcissism sometimes being referred to as *hypervigilant* narcissism. The vulnerable/neurotic aspect of narcissism is not represented in the NARC model because it intends to focus on the grandiose expression of narcissism. Recent research has shown that the vulnerable/neurotic aspect of narcissism often diverges from the assertive/extraverted and antagonistic/disagreeable aspects of narcissism in terms of its associations with attitudes and behaviors [57,58]. As a result, we believed it was important for us to distinguish between these three aspects of narcissism when considering the connections that narcissistic personality features had with exercise addiction through exercise-related motives. This approach is consistent with the recognition that it is important for researchers to distinguish between different aspects of narcissism in order to develop a more complete and nuanced understanding of the attitudes and behaviors that characterize narcissism.

**Figure 1:**
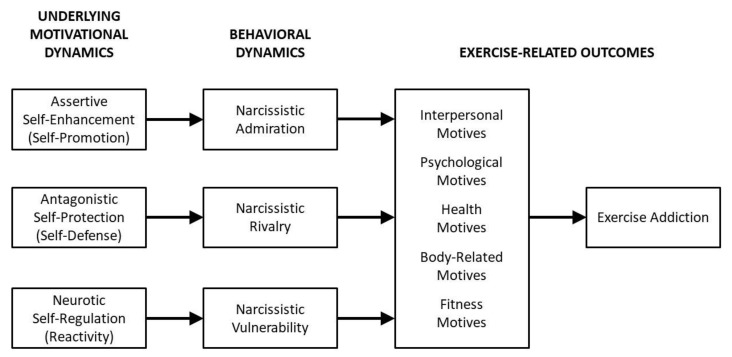
A modified version of the NARC model.

Exercise-related motives have been found to be associated with a range of attitudes and behaviors concerning exercise [47,59] as well as exercise addiction [60]. However, there are various approaches available for conceptualizing exercise-related motives [61,62]. The conceptualization of exercise-related motives that we adopted for the present study was developed by Markland and Ingledew [63]. This approach focuses on five higher-order exercise-related motives: *interpersonal motives* (which involve issues surrounding competition, social recognition, and affiliation), *psychological motives* (which deal with stress management, revitalization, and challenge), *health motives* (which reflect concerns with avoiding health problems, seeking positive health outcomes, and health-related pressures), *body-related motives* (which capture concerns revolving around physical appearance and weight management), and *fitness motives* (which involve issues such as the desire for strength, endurance, and agility). A model depicting the proposed associations that narcissistic personality features may have with exercise addiction through particular exercise-related motives is presented in Figure 1. We developed the following hypotheses for the present studies:

**Hypothesis** **1a.** *We expected narcissistic admiration to be positively associated with exercise addiction. The rationale for this prediction was that narcissistic admiration is characterized by the tendency to engage in self-promotion and self-enhancement which may contribute to the development of exercise addiction due to the benefits that exercise has for a range of outcomes including physical appearance, perceived formidability, and athletic performance. In essence, exercise may be appealing to individuals with elevated levels of narcissistic admiration because it provides them with an avenue to display their worth and value to others. This prediction is also consistent with the results of previous studies that have found narcissism to be associated with exercise addiction* [1,30].

**Hypothesis** **1b.** *We expected the positive association that narcissistic admiration had with exercise addiction to be due, at least in part, to exercise-related motives. That is, we expected narcissistic admiration to activate an array of motives concerning exercise that, in turn, would promote the development of exercise addiction. For example, narcissistic admiration has been shown to be linked with a strong desire for social recognition* [55] *so it seemed reasonable to assume that this aspect of narcissism may be associated with interpersonal motives for exercise that involve gaining recognition from others. It may be the satisfaction of these exercise-related motives—such as gaining recognition from others for one’s physical appearance—that facilitates the development of exercise addiction for individuals with high levels of narcissistic admiration*.

**Hypothesis** **2a.** *We expected narcissistic rivalry to be positively associated with exercise addiction. The rationale for this prediction was that narcissistic rivalry may be associated with exercise addiction due to a desire to outperform others. This hypothesis is consistent with previous research showing that narcissistic rivalry is characterized by a heightened sensitivity to competition as well as a desire to demonstrate superiority and dominance over others* [57,58]. *That is, exercise may be appealing to individuals with elevated levels of narcissistic rivalry because it provides them with an opportunity to display their dominance and superiority over others*.

**Hypothesis** **2b.** *We expected the positive association that narcissistic rivalry had with exercise addiction to be explained, at least in part, by the interpersonal motives for exercise. The basis for this prediction was that the interpersonal motives include issues such as outperforming others which would seem to align with the heightened sensitivity to competition that plays a central role in understanding the attitudes and behaviors that characterize narcissistic rivalry. We were uncertain about whether narcissistic rivalry would have indirect associations with exercise addiction through the other exercise-related motives because it was unclear whether individuals with high levels of narcissistic rivalry would be particularly motivated to engage in exercise due to issues such as improving their health or fitness. However, we examined these associations for exploratory purposes*.

**Hypothesis** **3a.** *We expected narcissistic vulnerability to be positively associated with exercise addiction. The rationale for this prediction was that narcissistic vulnerability is characterized by low self-esteem and the desire for external affirmation as well as the tendency to experience negative affect and psychological distress. It seems reasonable to assume these characteristics may contribute to the development of exercise addiction. In addition, narcissistic vulnerability is similar to the basic personality dimensions of neuroticism* [64] *which has been found to be associated with exercise addiction* [15,25,65,66].

**Hypothesis** **3b.** *We expected the psychological motives and body-related motives to mediate the association that narcissistic vulnerability had with exercise addiction. The rationale for this prediction was that narcissistic vulnerability is characterized by a reliance on external factors for self-esteem regulation* [42,53] *which would seem to be consistent with these particular exercise-related motives playing a role in the connection that this aspect of narcissism has with exercise addiction. We were uncertain whether narcissistic vulnerability would have indirect associations with exercise addiction through the other exercise-related motives, but we examined these associations for exploratory purposes*.

## 2. Materials and Methods

Participants were 2828 Israeli community members who responded to requests asking for volunteers to take part in an online study concerning “personality and attitudes about exercise” via postings on social media. Participation in this study was voluntary and participants provided their informed consent before completing the questionnaires. The appropriate sample size for this study was determined to be at least 250 participants based on a power analysis for the average effect size in social-personality psychology in conjunction with guidelines for reducing estimation error [67,68]. However, we deliberately employed oversampling in an effort to increase the statistical power of the study. More specifically, we used a time-based stopping rule for data collection that involved collecting data from as many participants as possible during a period of four months. Participants completed measures of narcissism, exercise-related motives, and exercise addiction via a secure website. To maintain equivalence of the instruments in the target language, all of the original questionnaires used in the present study were administered in Hebrew after being translated from the original English versions using the back-translation method. Data were excluded for 205 participants due to careless or inattentive responding: 48 participants were excluded for being univariate outliers, 17 participants were excluded for being multivariate outliers as assessed by Mahalanobis distance [69], 131 participants were excluded for having invariant response patterns as assessed by long-string analysis [70,71], and 3 participants were excluded due to inconsistent responding as assessed by inter-item standard deviation [72,73].

The final 2629 participants (1474 women and 1155 men) had a mean age of 30.22 years (*SD* = 11.43; range = 18–80). The mean body mass index of the final participants was 24.22 (*SD* = 4.30; range = 12.29–58.46 (Median = 23.53)) and they reported exercising an average of 2.21 times per week (*SD* = 1.86; range = 0–14 (Median = 2.00)) with 58% of participants reporting that they engaged in vigorous exercise at least once per week. Participants reported spending approximately 37% of their time exercising engaged in cardiovascular exercise and approximately 33% of their time exercising engaged in activities such as resistance training. The mean number of years of formal education for the final participants was 13.15 years (*SD* = 2.15; range = 10–30) and they were predominantly Jewish (95%) and heterosexual (90%). The self-reported current economic status of these participants was 13% “very good”, 46% “good”, 34% “moderate”, 6% “bad”, and 1% “very bad”.

### Measures

Narcissistic Admiration and Rivalry Questionnaire. We used the Narcissistic Admiration and Rivalry Questionnaire [54] to capture *narcissistic admiration* (9 items; e.g., “I manage to be the center of attention with my outstanding contributions” (α = 0.82)) and *narcissistic rivalry* (9 items; e.g., “I want my rivals to fail” (α = 0.85)). Participants were asked to rate how well each statement described them using scales that ranged from 1 (*not agree at all*) to 6 (*agree completely*). This instrument has been shown to possess adequate psychometric properties in previous studies [54].

Narcissistic Vulnerability Scale. We used the Narcissistic Vulnerability Scale [74] to capture the vulnerable aspect of narcissism. The Narcissistic Vulnerability Scale consists of 11 adjectives (e.g., “Self-absorbed”, “Fragile”, “Underappreciated” (α = 0.82)) that participants were asked to rate with regard to how well each described them using scales that ranged from 1 (*not at all*) to 7 (*extremely*). The Narcissistic Vulnerability Scale has been shown to possess adequate psychometric properties in previous studies [74].

Exercise Motivations Inventory. We used the Exercise Motivations Inventory-2 [63] to capture the following motives for engaging in exercise: *interpersonal motives* (12 items; e.g., “Personally, I exercise [or might exercise] to show my worth to others” (α = 0.92)), *psychological motives* (17 items; e.g., “Personally, I exercise [or might exercise] because it makes me feel good” (α = 0.94)), *health motives* (11 items; e.g., “Personally, I exercise [or might exercise] because it makes me feel good to prevent health problems” (α = 0.89)), *body-related motives* (8 items; e.g., “Personally, I exercise [or might exercise] to help control my weight” (α = 0.90)), and *fitness motives* (8 items; e.g., “Personally, I exercise [or might exercise] to get stronger” (α = 0.92)). Participants were asked to rate how well each statement described them using scales that ranged from 1 (*not at all true for me*) to 6 (*very true for me*). This instrument has been shown to possess adequate psychometric properties in previous studies [63].

Exercise Addiction Inventory. We used the Exercise Addiction Inventory [17] to capture the extent to which participants were addicted to exercise (6 items; e.g., “Exercise is the most important thing in my life” (α = 0.79)). Participants were asked to rate their level of agreement with each statement using scales that ranged from 1 (*strongly disagree*) to 5 (*strongly agree*). This instrument has been shown to possess adequate psychometric properties in previous studies [17].

## 3. Results

Descriptive statistics and zero-order correlations are presented in Table 1. Narcissistic admiration had small-to-medium positive correlations with narcissistic rivalry, narcissistic vulnerability, interpersonal motives, psychological motives, health motives, body-related motives, fitness motives, and exercise addiction. Narcissistic rivalry had a large positive correlation with narcissistic vulnerability as well as small-to-medium positive correlations with interpersonal motives, health motives, body-related motives, fitness motives, and exercise addiction. Narcissistic rivalry also had a small *negative* correlation with psychological motives. Narcissistic vulnerability had small positive correlations with interpersonal motives, body-related motives, fitness motives, and exercise addiction. In addition, narcissistic vulnerability had a small *negative* correlation with psychological motives and it was not correlated with health motives.

Parallel multiple mediation. We were interested in the possibility that the associations the narcissistic personality features had with exercise addiction may be due, at least in part, to exercise-related motives. To examine this possibility, we conducted a parallel multiple mediation analysis using Model 4 of the PROCESS macro [75]. One advantage of this parallel multiple mediation analysis is that it allowed us to examine whether the indirect association that an aspect of narcissism (e.g., narcissistic admiration) had with exercise addiction through a particular mediator (e.g., interpersonal motives) emerged when statistically controlling for the other aspects of narcissism and exercise-related motives that were included in the same model. Direct and indirect effects were estimated using a bootstrap resampling process that was repeated 10,000 times in order to generate 95% bootstrap confidence intervals. All variables were standardized prior to analysis in order to enhance the interpretability of the coefficients. The Variance Inflation Factor (VIF) values for this analysis were less than 1.92 which suggests that multicollinearity was not an issue [76].

**Figure 2:**
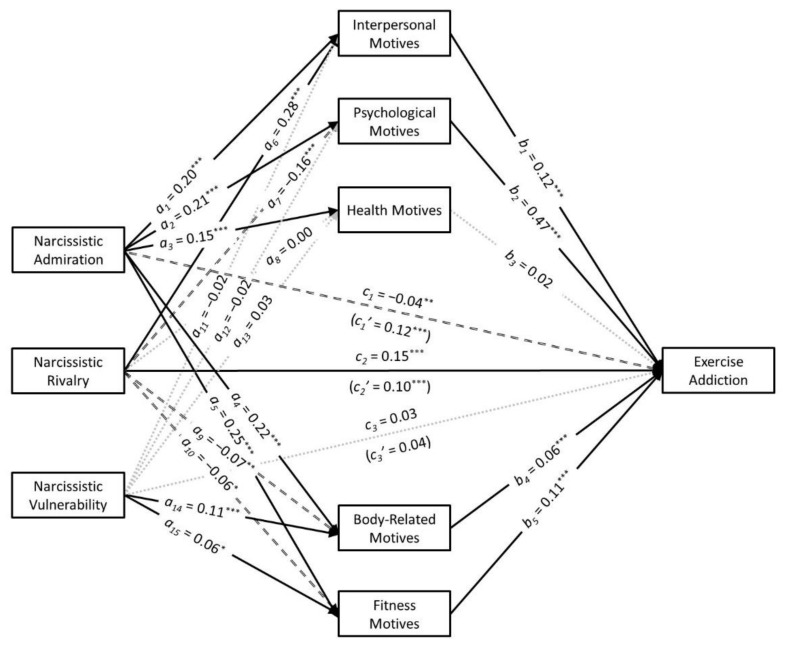
The results of the parallel multiple mediation analyses with the exercise-related motives mediating the associations that narcissistic admiration, narcissistic rivalry, and narcissistic vulnerability had with exercise addiction. *Note:* The total effects (i.e., c_1′_, c_2′_, and c_3′_) are presented in parentheses. The significant positive associations are indicated by solid black arrows. The significant negative associations are indicated by dashed black arrows. The dotted gray lines represent nonsignificant associations. * *p* < 0.05; ** *p* < 0.01; *** *p* < 0.001.

The results of this parallel multiple mediation analysis are presented in Figure 2. These results revealed that narcissistic admiration had small positive unique associations with each of the exercise-related motives: interpersonal motives (*a*_1_ = 0.20, *t* = 10.24, *p* < 0.001, *CI*_95%_ [0.16, 0.24]), psychological motives (*a*_2_ = 0.21, *t* = 9.92, *p* < 0.001, *CI*_95%_ [0.17, 0.25]), health motives (*a*_3_ = 0.15, *t* = 6.95, *p* < 0.001, *CI*_95%_ [0.11, 0.19]), body-related motives (*a*_4_ = 0.22, *t* = 10.80, *p* < 0.001, *CI*_95%_ [0.18, 0.26]), and fitness motives (*a*_5_ = 0.25, *t* = 12.04, *p* < 0.001, *CI*_95%_ [0.21, 0.29]). In contrast, narcissistic rivalry had a small positive association with interpersonal motives (*a*_6_ = 0.28, *t* = 11.78, *p* < 0.001, *CI*_95%_ [0.23, 0.32]) but small negative associations with psychological motives (*a*_7_ = −0.16, *t* = −6.54, *p* < 0.001, *CI*_95%_ [−0.21, −0.11]), body-related motives (*a*_9_ = −0.08, *t* = −3.07, *p* = 0.002, *CI*_95%_ [−0.12, −0.03]), and fitness motives (*a*_10_ = −0.06, *t* = −2.32, *p* = 0.02, *CI*_95%_ [−0.11, −0.01]). Narcissistic rivalry was not associated with health motives (*a*_8_ = 0.00, *t* = −0.08, *p* = 0.94, *CI*_95%_ [−0.05, 0.05]). Narcissistic vulnerability had small positive associations with body-related motives (*a*_14_ = 0.11, *t* = 4.77, *p* < 0.001, *CI*_95%_ [0.07, 0.16]) and fitness motives (*a*_15_ = 0.06, *t* = 2.42, *p* = 0.02, *CI*_95%_ [0.01, 0.10]) but was not associated with interpersonal motives (*a*_11_ = −0.02, *t* = −1.06, *p* = 0.29, *CI*_95%_ [−0.07, 0.02]), psychological motives (*a*_12_ = −0.02, *t* = −0.75, *p* = 0.45, *CI*_95%_ [−0.06, 0.03]), or health motives (*a*_13_ = 0.03, *t* = 1.07, *p* = 0.28, *CI*_95%_ [−0.02, 0.07]). In turn, interpersonal motives (*b*_1_ = 0.12, *t* = 6.45, *p* < 0.001, *CI*_95%_ [0.08, 0.16]), psychological motives (*b*_2_ = 0.47, *t* = 23.92, *p* < 0.001, *CI*_95%_ [0.43, 0.51]), body-related motives (*b*_4_ = 0.06, *t* = 3.42, *p* < 0.001, *CI*_95%_ [0.02, 0.09]), and fitness motives (*b*_5_ = 0.11, *t* = 5.66, *p* < 0.001, *CI*_95%_ [0.07, 0.15]) had small-to-medium positive associations with exercise addiction. Health motives did not have a unique association with exercise addiction (*b*_3_ = 0.02, *t* = 1.08, *p* = 0.28, *CI*_95%_ [−0.01, 0.05]).

Tests of mediation found that narcissistic admiration had positive indirect associations with exercise addiction through interpersonal motives (*a*_1_*b*_1_ = 0.02, *z* = 5.44, *p* < 0.001, *CI*_95%_ [0.02, 0.03]), psychological motives (*a*_2_*b*_2_ = 0.10, *z* = 9.16, *p* < 0.001, *CI*_95%_ [0.08, 0.12]), body-related motives (*a*_4_*b*_4_ = 0.01, *z* = 3.25, *p* = 0.001, *CI*_95%_ [0.01, 0.02]), and fitness motives (*a*_5_*b*_5_ = 0.03, *z* = 5.11, *p* < 0.001, *CI*_95%_ [0.02, 0.04]). In contrast, narcissistic rivalry had a positive indirect association with exercise addition through interpersonal motives (*a*_6_*b*_1_ = 0.03, *z* = 5.64, *p* < 0.001, *CI*_95%_ [0.02, 0.05]) but negative indirect associations with exercise addiction through psychological motives (*a*_7_*b*_2_ = −0.08, *z* = −6.30, *p* < 0.001, *CI*_95%_ [−0.10, −0.05]), body-related motives (*a*_9_*b*_4_ = −0.01, *z* = −2.23, *p* = 0.03, *CI*_95%_ [−0.01, 0.00]), and fitness motives (*a*_10_*b*_5_ = −0.01, *z* = −2.12, *p* = 0.03, *CI*_95%_ [−0.01, 0.00]). Narcissistic vulnerability had positive indirect associations with exercise addiction through body-related motives (*a*_14_*b*_4_ = 0.01, *z* = 2.74, *p* = 0.006, *CI*_95%_ [0.00, 0.01]) and fitness motives (*a*_15_*b*_5_ = 0.01, *z* = 2.20, *p* = 0.03, *CI*_95%_ [0.00, 0.01]).

**Figure 3:**
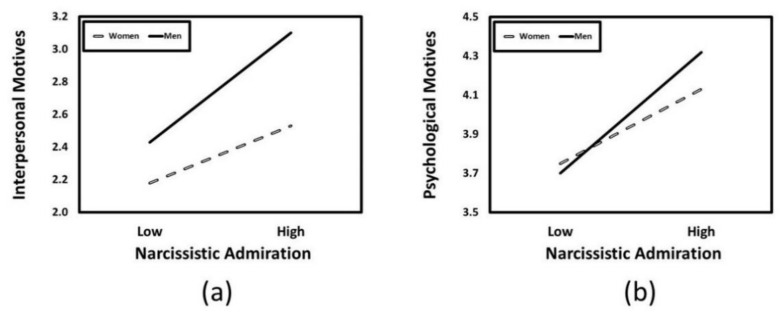
Predicted values illustrating the interaction of narcissistic admiration (at values that are one standard deviation above and below its mean) and gender for interpersonal motives (**a**) and psychological motives (**b**).

Moderated mediation. Previous research has revealed that men consistently report higher levels of narcissism than women [77] and gender has sometimes been shown to moderate the associations that narcissistic personality features have with certain outcomes [78]. As a result, we conducted an exploratory analysis to examine whether gender moderated the strength of the indirect associations that narcissistic personality features had with exercise addiction through exercise-related motives. This exploratory moderated mediation analysis was conducted using Model 8 of the PROCESS macro [75]. Results indicated that gender did not moderate the indirect associations that narcissistic admiration, narcissistic rivalry, or narcissistic vulnerability had with exercise addiction through any of the exercise-related motives. Although gender did not moderate the indirect associations that narcissistic personality features had with exercise addiction, it did moderate the associations that narcissistic admiration had with interpersonal motives (*β* = 0.07, *t* = 3.38, *p* < 0.001, *CI*_95%_ [0.03, 0.10]) and psychological motives (*β* = 0.05, *t* = 2.47, *p* = 0.01, *CI*_95%_ [0.01, 0.09]). The predicted values for the narcissistic admiration × gender interaction for interpersonal motives are presented in Figure 3a. We supplemented this analysis with the simple slopes recommended for describing interactions that involve a continuous predictor [79]. Simple slopes tests revealed that narcissistic admiration was positively associated with interpersonal motives for women (*β* = 0.14, *t* = 4.95, *p* < 0.001, *CI*_95%_ [0.08, 0.20]) but that this positive association was significantly stronger for men (*β* = 0.27, *t* = 10.22, *p* < 0.001, *CI*_95%_ [0.22, 0.32]). The predicted values for the narcissistic admiration × gender interaction for psychological motives are presented in Figure 3b. Similar to the results for interpersonal motives, simple slopes tests revealed that narcissistic admiration was positively associated with psychological motives for women (*β* = 0.15, *t* = 5.04, *p* < 0.001, *CI*_95%_ [0.09, 0.21]) but that this association was particularly strong for men (*β* = 0.26, *t* = 8.98, *p* < 0.001, *CI*_95%_ [0.20, 0.31]). These patterns reveal that narcissistic admiration was positively associated with interpersonal motives and psychological motives for both women and men but that these associations were especially strong for men.

## 4. Discussion

The purpose of the present research was to examine whether exercise-related motives mediated the associations that narcissistic personality features had with exercise addiction. We found partial support for our predictions. For example, our results showed that each aspect of narcissism was positively correlated with exercise addiction as we expected. However, it is important to note that these associations were small in magnitude and that narcissistic vulnerability did not have a unique association with exercise addiction when narcissistic admiration and narcissistic rivalry were included in the same analysis. This suggests that the residual form of narcissistic vulnerability that remains after its overlap with narcissistic admiration and narcissistic rivalry has been statistically removed does not have a direct association with exercise addiction. One possible explanation for this pattern is that the zero-order correlation that narcissistic vulnerability had with exercise addiction may have been due to the common “core” of narcissism that is shared with narcissistic admiration and narcissistic rivalry rather than being specific to this aspect of narcissism.

Despite the associations that narcissistic personality features had with exercise addiction being weaker than we anticipated, each aspect of narcissism had indirect associations with exercise addiction through particular exercise-related motives. For example, narcissistic admiration and narcissistic rivalry had similar positive indirect associations with exercise addiction through interpersonal motives. These indirect associations were consistent with our predictions and suggest that issues pertaining to competition and social recognition likely play important roles in the connections that these aspects of narcissism have with exercise addiction. That is, narcissistic individuals may be so focused on the interpersonal rewards that are involved with increasing levels of exercise (e.g., outperforming others at the gym) that they fail to recognize the potential costs that are involved with allowing exercise to shape so much of their lives. In essence, narcissistic individuals may use exercise as a means of affirming their value and worth. However, this may unintentionally create an escalating pattern for narcissistic individuals in which their increasingly desperate pursuit of self-worth through exercise makes it even more difficult for them to feel satisfied and secure, resulting in an addiction to exercise. These results align with recent arguments that attitudes and behaviors connected with the pursuit of status may be a central feature of narcissism [26,55,80] because exercise may serve as another means for narcissistic individuals to navigate local status hierarchies by demonstrating their dominance and superiority over others.

Narcissistic admiration and narcissistic rivalry had similar associations with interpersonal motives, but it is important to note that they had divergent indirect associations with exercise addiction through psychological motives, body-related motives, and fitness motives. More specifically, these indirect associations were positive for narcissistic admiration but negative for narcissistic rivalry. This suggests that the motivational profiles concerning exercise may be quite different for narcissistic admiration and narcissistic rivalry. However, it is important to note that narcissistic rivalry actually had positive zero-order correlations with each of these motives and that these associations only reversed their sign and became negative associations when the other aspects of narcissism were included in the same analysis. It is not unusual for the association that an aspect of narcissism has with an outcome to be altered by the inclusion of other aspects of narcissism in the same analysis. These sorts of patterns are sometimes referred to as *suppression effects* [81] and they can involve changes in the magnitude of associations or even changes in the direction of associations, as was observed in the present study. Taken together, these results reveal that narcissistic admiration has clear positive associations with psychological motives, body-related motives, and fitness motives which, in turn, are associated with exercise addiction. In contrast, the nature of the associations that narcissistic rivalry had with these particular exercise-related motives was found to depend on whether the other aspects of narcissism were included in the analysis. Future research should attempt to gain a better understanding of the associations that narcissistic rivalry has with psychological motives, body-related motives, and fitness motives.

Narcissistic vulnerability had positive indirect associations with exercise addiction through body-related motives and fitness motives that were similar to those observed for narcissistic admiration. This suggests that individuals with elevated levels of narcissistic vulnerability may be motivated to engage in exercise because it provides them with an opportunity to affirm their worth and value in the eyes of others. This is consistent with previous results showing that narcissistic vulnerability is characterized by a reliance on external factors such as physical appearance for self-esteem regulation [42]. These results also align with findings from past studies showing narcissistic vulnerability to be a risk factor for an array of concerns related to body image [82,83]. Taken together, these results suggest that appearance-related concerns likely play an important role in the connection between narcissistic vulnerability and exercise addiction.

We also conducted exploratory analyses that examined whether gender moderated the associations that narcissistic personality features had with exercise-related motives and exercise addiction. These analyses revealed that narcissistic admiration was positively associated with interpersonal motives and psychological motives for both women and men but that these associations were especially strong for men. It is possible that these associations may be particularly pronounced for men because issues surrounding perceived masculinity are often linked with physical fitness [84]. This pattern is consistent with the results of other recent studies showing that men may be particularly susceptible to some problematic attitudes and behaviors concerning exercise [85,86,87,88].

The results of the present studies provide additional support for the importance of distinguishing between different aspects of narcissism. There were certainly similarities for these narcissistic personality features (e.g., each had small positive correlations with exercise addiction) but there were also important differences between these aspects of narcissism. For example, the indirect associations that narcissistic rivalry had with exercise addiction through some of the exercise-related motives were quite different than those that emerged for narcissistic admiration and narcissistic vulnerability. Furthermore, narcissistic rivalry had competing indirect associations with exercise addiction through the exercise-related motives (i.e., a positive indirect association through the interpersonal motives but negative indirect associations through the psychological motives, body-related motives, and fitness motives). The complexity of these associations would have been obscured if we had not distinguished between these aspects of narcissism. This suggests that future studies concerning the associations between narcissism and exercise-related constructs should continue to distinguish between these aspects of narcissism in order to allow for a more complete and nuanced understanding of these connections.

The present study provides additional insights into the connections that narcissism has with exercise-related motives and exercise addiction, but it would be beneficial for future studies to expand on these results. For example, it would be helpful to examine whether exercise-related motives may shed light on the associations that narcissism has with other attitudes and behaviors surrounding exercise such as *exercise commitment*. This may be informative because previous research has shown narcissistic personality features to be associated with elevated levels of exercise behaviors [40] as well as appearance-related concerns [41,42]. It is possible that exercise-related motives—especially interpersonal motives for exercise—may provide a better understanding of how narcissistic personality features are able to “get outside the skin” [89] and produce particular patterns of behaviors and attitudes related to exercise.

It is important to note that we focused on “exercise addiction” even though there has been considerable debate about whether it should actually be considered as an *addiction* [1]. However, it has been argued that it is appropriate to refer to this issue as exercise *addiction* because it involves compulsive tendencies and feelings of dependence, which are not commonly observed among those who are simply committed to exercise [9]. Another potential concern is that exercise addiction is not included as a disorder in the *DSM-5* [22]. We decided to focus on exercise addiction because we believe it warrants empirical attention regardless of the debate regarding whether it should be included in future editions of the *DSM*. This is important because research concerning exercise addiction may help inform decisions regarding whether it warrants inclusion in future editions of the *DSM*.

The present study had a number of strengths (e.g., multidimensional view of narcissism, large sample of community members) but it is also important to acknowledge some of its potential limitations. The most important limitation is that we cannot establish the direction of causality between narcissism, exercise-related motives, and exercise addiction due to the correlational nature of the present study. We adopted a process model that was based on the idea that personality traits often activate motives which are then satisfied by adopting certain attitudes or engaging in particular behaviors. Although the present results were largely consistent with the expected indirect associations, this does not necessarily demonstrate the causal pattern implied by the use of a mediational analysis because it is entirely possible that other causal patterns may actually exist between these variables. For example, it is possible that exercise-related motives may actually influence the development of narcissistic personality features rather than these motives being consequences of narcissism. It would be beneficial for future research to use experimental designs or longitudinal studies in order to gain a better understanding of the potential causal links between these variables.

Another limitation is that we relied exclusively on self-report instruments for the present study. As a consequence, it is possible that our results may have been influenced by issues such as individuals engaging in socially desirable responding (e.g., individuals being reluctant to acknowledge symptoms of exercise addiction in order to avoid portraying themselves negatively) or having limited insights into certain aspects of their psychological processes (e.g., individuals not really understanding their own motives for engaging in exercise). It would be beneficial for future research concerning narcissism and exercise to avoid relying exclusively on self-report instruments (e.g., including direct behavioral measures of exercise-related behaviors). The final limitation is that we relied on instruments that were translated into Hebrew for the present study. We followed the back-translation method which is commonly used in similar situations [90] but there are some limitations to this particular translation method [91]. Although the back-translation method is an extensively used translation method, it would be helpful if future studies attempted to replicate the present results using other instruments that were intended for use with those who speak Hebrew. Despite these limitations, the present study extends what is known about the links between narcissism, exercise-related motives, and exercise addiction.

## 5. Conclusions

The present study examined the associations that narcissistic admiration, narcissistic rivalry, and narcissistic vulnerability had with exercise addiction as well as whether these associations were mediated by exercise-related motives. Our results showed that each aspect of narcissism had small positive associations with exercise addiction. Narcissistic admiration and narcissistic rivalry had similar positive indirect associations with exercise addiction through interpersonal motives, but they had divergent indirect associations with exercise addiction through psychological motives, body-related motives, and fitness motives. Narcissistic vulnerability had positive indirect associations with exercise addiction through body-related motives and fitness motives that were similar to those observed for narcissistic admiration. These results suggest that exercise-related motives may play important roles in the associations that narcissistic personality features have with exercise addiction.

## Figures and Tables

**Table 1:** Intercorrelations and descriptive statistics.

	1	2	3	4	5	6	7	8	9
1. Narcissistic Admiration	—								
2. Narcissistic Rivalry	0.36 ***	—							
3. Narcissistic Vulnerability	0.06 **	0.55 ***	—						
4. Interpersonal Motives	0.30 ***	0.34 ***	0.14 ***	—					
5. Psychological Motives	0.15 ***	−0.10 ***	−0.10 ***	0.42 ***	—				
6. Health Motives	0.15 ***	0.07 **	0.03	0.22 ***	0.29 ***	—			
7. Body-Related Motives	0.20 ***	0.07 **	0.08 ***	0.16 ***	0.25 ***	0.34 ***	—		
8. Fitness Motives	0.23 ***	0.06 **	0.04 *	0.38 ***	0.55 ***	0.40 ***	0.41 ***	—	
9. Exercise Addiction	0.16 ***	0.16 ***	0.10 ***	0.42 ***	0.58 ***	0.25 ***	0.25 ***	0.45 ***	—
Mean	3.36	1.90	2.60	2.53	3.97	3.45	4.11	3.88	2.54
Standard Deviation	0.99	0.84	0.98	1.22	1.20	1.12	1.37	1.40	0.94

* *p* < 0.05; ** *p* < 0.01; *** *p* < 0.001.

## Data Availability

The data presented in this study are openly available on the Open Science Framework (OSF) at https://osf.io/mtczy/ (Accessed date: 12 March 2021).
